# Replies to Fratantonio and Lasonen-Aarnio; Goldberg; Greco; Kelp, Carter and Simion; Littlejohn; and Williamson

**DOI:** 10.1007/s11098-022-01813-6

**Published:** 2022-04-05

**Authors:** Jessica Brown

**Affiliations:** University of St Andrews, St Andrews, UK

Replies

I want to thank all the contributors for their thoughtful comments on the book, Brown (2018). I’ve tried to respond to as many of their points as possible within the space available.

## Fratantonio and Lasonen-Aarnio

Fratantonio and Lasonen-Aarnio (henceforth FLA) argue that an infallibilist can evade two of my main objections. The first of these objections was my claim that infallibilism can avoid scepticism about important forms of knowledge only by endorsing SKSS: if S knows that p, then p is part of S’s evidence for p. But, as I pointed out, it is standardly infelicitous to cite a proposition as evidence for itself.

Following Fratantonio (2021) FLA suggest that SKSS admits of two different readings, of which only one would give rise to problematic infelicity data but that reading needn’t be accepted by the infallibilist. First consider a motivational reading, where q is motivating evidence for p for S if and only if q is the evidence that motivated S to believe that p. In this sense, if S is asked what’s her evidence for p, it would be infelicitous for her to reply by saying p. For, p is not what motivated her to believe p which is plausibly instead motivated by some distinct evidence. However, they think that when “evidence for” is understood in a justifying way, it doesn’t give rise to any infelicity data so that the infallibilist can accept this second reading of SKSS. p is justifying evidence for p for S if and only if p is the evidence in virtue of which S has justification to believe p.

In reply, it seems to me that even the second justifying reading of SKSS also gives rise to infelicity data. When one asks for the evidence for p in this second sense, one is asking for evidence which provides justification to believe p, or reason to believe p. But, intuitively, p doesn’t provide justification or reason to believe that p. For instance, I might be interested in the substantive question of whether the government lifted coronavirus restrictions too early. Knowing that my friend believes that’s so, I might ask, “What’s your evidence that the government lifted restrictions too early?” Here it would be infelicitous for my friend to reply to my question by merely restating that the government lifted coronavirus restrictions too early. Thus, whether we are interested in the motivating or justifying sense of “evidence for p” it seems infelicitous to cite p as evidence for p.

Setting that aside, FLA suggest that the infallibilist needn’t accept SKSS even understood to involve the notion of justifying evidence for p. Relatedly, they suggest that the formulation of infallibilism I employ doesn’t accurately characterise certain positions. Their argument for these points rests on the idea that one might come to embrace infallibilism as a result of commitment to a recognisably fallibilist view of knowledge and the sufficiency of knowledge for evidence (SKE). The relevant fallibilist view is an explanationist account of evidential support on which S’s evidence e provides evidential support for p if and only if p is the best sufficiently good or plausible explanation for why S has e. Since p might provide the best sufficiently good explanation of why S has e even if e doesn’t entail that p, this looks like a fallibilist position. But, FLA argue that when combined with SKE it entails infallibilism*, so would be wrongly classified as an infallibilist position. Furthermore, they argue that this style of infallibilism wouldn’t entail SKSS for it’s implausible that a proposition p is the best sufficiently good explanation for p.

Let’s examine the suggestion more carefully. Suppose that the explanationist grants that S knows by inference to the best explanation that the dog ate the birthday cake, or d, since d is the best explanation of S’s evidence, namely the missing cake (c), the dog’s guilty look (g), together with facts about the location of the cake and the dog (l). So far, we needn’t suppose that S’s evidence for the claim that the dog ate the birthday cake, namely {c, g, l}, entails that the dog ate the birthday cake. Do things change if we add SKE? Of course, if S’s evidence now includes d, then her evidence does entail d. But to show that this position amounts to infallibilism* we need to establish the further claim that her *evidence for* d entails that d. (Recall: infallibilism* states that one can know that p only if one's evidence for p entails that p.) But there seems no reason for the fallibilist explanationist to accept that S’s *evidence for* d has expanded to include d. Indeed, she has reason not to accept this. If whenever a subject knows that p, p is treated as part of the subject’s evidence for p, then we cannot say that some known claims have better or stronger evidential support than others. But given that the explanationist holds that it’s sufficient for S to know that p that p provides the best sufficiently good or plausible explanation for why S has the evidence, it’s part and parcel of the view that some explanations are better than others and so some claims are better evidentially supported than others. If, as I suggest, the explanationist position isn’t plausibly committed to infallibilism*, then its relation to SKSS is irrelevant to whether infallibilism is committed to SKSS.

FLA then turn to consider my arguments in defence of defeat. In particular, they question my argument against level-splitting views on which a subject can rationally be in an epistemically akratic state, such as believing that p while also believing that her evidence does not support that p. Against such views, I argue that they sanction certain kinds of bad reasoning. FLA worry that this problem is faced not only by level-splitters but also by fallibilists. They provide an example in which S’s evidence both supports that p and that she has evidence that her evidence does not support that p. If a fallibilist were to endorse the evidentialist view on which one has justification to believe what is likely on one’s evidence, S has justification to believe both p and that she has evidence that her evidence does not support that p. But, part of my argument against level-splitting views is that if such a combination of beliefs is justified, it will lead to blatantly bad reasoning. So, they question how a fallibilist can avoid the kind of bad reasoning I argue affects level-splitting views. In reply, and as I argue in the book, the fallibilist should reject the relevant evidentialist view and endorse certain inter-level coherence requirements which require coherence between the attitudes she takes at first-order and the attitudes she has at higher-levels. While the book mentioned coherence requirements between first-order beliefs and beliefs about what one’s evidence supports, for similar reasons one might defend coherence requirements between first-order states and beliefs about what one’s evidence supports about what one’s evidence supports. This would deal with the problematic examples and would seem natural from the point of view of somebody already committed to inter-level coherence principles.

## Goldberg

While Goldberg agrees with much of my case against infallibilism, he thinks that the case of testimonial knowledge may not present such a problem for infallibilism as I suggested. I argued that testimony presents a problem because it is one of several kinds of knowledge where what is intuitively one’s evidence for a known claim does not entail what is known. In reply, Goldberg suggests a more generous view of testimonial evidence which is supposed to help infallibilism.

Goldberg suggests that when all goes well, testimony constitutes a certain kind of epistemic link to the facts, where a subject S is epistemically linked to the fact that p when she truly believes that p and her belief that p was acquired in a way W and in conditions C such that, in C, true beliefs acquired through W constitute knowledge. The claim that testimony can constitute such an epistemic link doesn’t in itself say anything about testimonial evidence. However, Goldberg suggests that this general approach can be developed in a variety of epistemological frameworks in such a way that it does make claims about testimonial evidence. First, he suggests it might be combined with Dretske’s notion of a conclusive reason, on which a reason r is a conclusive reason for S to believe that p just in case S wouldn’t have r unless p. But even if testimony can, when things go well, provide a conclusive reason in this sense, the reason doesn’t entail what is known. To say that S would not have testified that p unless p, is not to say that there is no possible situation in which S testifies falsely that p.

Now consider Goldberg’s other suggestion, an appeal to “extended evidentialism” according to which a hearer’s testimonial evidence for p includes “not just the fact that a (seemingly sincere and competent) speaker says so but also the facts that constitute S’s relevant evidence—the evidence on the basis of which S said so”. On this view, when a hearer, H, gains knowledge that p from the testimony of a speaker, S, whether H has evidence which entails p depends on whether S does. For instance, suppose that Watson gains knowledge that p from the testimony of Holmes, where Holmes doesn’t tell Watson what his evidence for p is. On extended evidentialism, whether Watson has evidence that entails that p depends on whether Holmes does. Holmes might have deduced p from known premises; but, alternatively, he might have come to know p by inference to the best explanation from premises which do not entail p. Either way, testimony doesn’t provide any additional problem for infallibilism. Rather, the core question is whether knowledge other than testimony meets the infallibilist criterion.

However, while appeal to extended evidentialism would in this way show that testimony doesn’t provide any additional problem for infallibilism, it’s a highly controversial view. It’s in tension with the widely held view that a proposition is part of S’s evidence only if it is part of her epistemic perspective (for recent discussion, see Fassio, 2021). For instance, many claim that p is part of S’s evidence only if S believes, or perhaps knows, that p. Some embrace a slightly more generous account of evidence on which one’s evidence also includes what one is in a position to know, where one is in a position to know that p if one can know it on the basis of evidence one already has. But, extended evidentialism allows that a hearer’s evidence can include propositions which are not part of the hearer’s epistemic perspective in this sense. Relatedly, extended evidentialism is in tension with links between a subject’s evidence and our normative expectations of her. If a subject has evidence which obviously supports a proposition, p, which is highly relevant to her practical interests, we would expect the subject to believe that p and act in the light of that information. But, these connections between evidence and normative expectations would be broken on the extended evidence view.

I conclude that testimony still provides a difficult challenge for infallibilism.

## Greco

Greco focuses on my claim that infallibilism is committed to SKSS: if one knows that p, then p is part of one’s evidence for p. But, that’s problematic since it’s typically infelicitous to cite a known proposition as evidence for itself. In reply, Greco argues that this infelicity problem cannot be avoided by embracing fallibilism rather than infallibilism for it arises from the probability raising account of evidential support (PR: e is evidence for h for S if and only if S’s evidence includes e and P(h/e) > P(h)). Instead, his preferred solution is to combine the probability raising conception with contextualism about evidence according to which, when we raise the question of the evidence for p, p is no longer evidence. Thus, a known proposition is not evidence for itself.

In reply, I first want to note that setting aside the relevant probabilistic conception of evidence, fallibilism and infallibilism stand in a different dialectical relationship to SKSS. As I argue in chapter 2, infallibilism is best understood as holding that knowing that p not only requires evidence which entails that p, but also that one has *evidence for* p which entails that p. But, then, in order to avoid scepticism about large swathes of what we ordinarily take ourselves to know, the infallibilist needs to endorse SKSS. By contrast, since the fallibilist allows that one can know that p even if one doesn’t have evidence for p which entails that p, she is not committed to SKSS.

However, even granting that, it may be pointed out that if we do assume the relevant probabilistic conception of evidential support, then a version of the problem of self-support faces any (non-contextualist) view of evidence. For instance, restricting one’s evidence to what one knows in some particular kind of way, say what one non-inferentially knows, would still face an analogous problem. I agree that that’s so, and indeed argued at length that restricting what counts as our evidence is no solution to the problem of evidential self-support in an earlier article (Brown, 2015, pp. 44–45). So Greco is wrong to suppose that my preferred solution to the problem involves “granting the sufficiency of evidence for self-support, and attempting to avoid the infelicity by adopting a narrower conception of evidence than that favoured by infallibilists”. The passage he takes to show me to be committed to this strategy in fact has an entirely different argumentative purpose.

If the solution is not to restrict what counts as one’s evidence, what is the solution? Broadly, there are the following three options:

1. Accept PR and that any proposition which is part of one’s evidence is evidence for itself but offer a pragmatic or error-theoretic explanation of why it is often infelicitous to cite a proposition as evidence for itself. (e.g. Williamson, 2000)
2. Accept PR but hold a contextualist account of evidence on which when we’re interested in the question of what is the evidence for p, p is no longer part of our evidence. Thus, it’s always false to cite a proposition as evidence for itself. (Greco)
3. Reject PR or modify it so that it doesn’t have the consequence that any proposition which is part of one’s evidence is evidence for itself.

Greco agrees with the argument of the book that the first option fails. Greco himself favours the second option (2), but I considered and criticised that option in Brown (2015). A first worry is that the proposed contextualist view makes it too easy to lose evidence. In particular, if one starts out knowing that p so that p is part of one’s evidence, one can lose p as evidence merely if somebody inquires into one’s evidence for p without providing any evidence against p. Second, the proposed contextualist solution doesn’t fit the shape of the intuitive data. In particular, the intuitive data seems to be that even when a proposition is evidence, it is not evidence for itself. By contrast, the contextualist offers an explanation of how some proposition isn’t evidence at all for anything in certain contexts. As a result, the proposed explanation fails to explain why conjunctive claims of the following kinds are perfectly in order: “That the vaccine is safe is evidence that Pfizer’s shares will rise, but isn’t evidence that the vaccine is safe”. Given these problems, Brown (2015) argued that we should adopt a version of the last option, rejecting the probability raising conception of evidential support. In doing so, I agree with Greco, and indeed argued, that there is no simple formal fix such as adding the condition that a proposition isn’t evidence for itself. For, no such simple fix is likely to deal with all the cases (52–53). Instead, I suggested that we should use the role of evidence in enquiry in developing a better account of evidential support. One of the central roles of evidence in enquiry is to enable us to gain, for the first time, justified belief in claims or, if we already have such justification, to strengthen it. As it is sometimes put, a central role of evidence in enquiry is “warrant transmission”. I suggested, then, that we should either supplement the probability raising account with a warrant transmission condition, or perhaps instead abandon the probability raising account and employ the notion of warrant transmission in explaining evidential support.

## Kelp, Carter and Simion. (KCS)

KCS raise a range of issues in the comments. In their first set of comments, they aim to defend three key commitments of contemporary infallibilism, viz. factivity of evidence (if p is part of one’s evidence, then p is true); SKE (if one knows that p, then p is part of one’s evidence); and SKSS (if one knows that p, then p is evidence for p). First, they raise the concern that what they take to be my objection to SKE would apply to any potential fallibilist account of evidence. They take me to object to SKE on the grounds that, even if one knows that p, it’s not felicitous to reply to a request for evidence for p by saying “p”. But that’s to mistake my argument. I use the relevant infelicity data not against SKE, but against SKSS. When one is asked for “evidence for p” one is being asked for evidence which supports that p, not for any part of one’s evidence whatsoever. In itself, an account of evidence, whether fallibilist or not, is silent on when a piece of evidence supports a hypothesis.

Turning to SKSS, they suggest that it’s wrong to claim as I do that the infallibilist needs to endorse SKSS to avoid scepticism about important kinds of knowledge, such as knowledge by inference to the best explanation, enumerative induction, and testimony. They suggest that “while data from testimony, inference to the best explanation and enumerative induction may not entail what is known, they may when combined with a sufficiently friendly epistemic environment”. It’s not clear how appeal to a friendly epistemic environment will help. To illustrate, suppose that I acquire knowledge that p from the testimony of a speaker who says that “p”. That she said that p doesn’t entail that p, even with the addition of the claims that speakers are generally reliable, or that she is generally reliable. So, even adding that the testimony takes place in a friendly epistemic environment, the testimony doesn’t entail what’s known. (Of course, that she said that p combined with the claim that every assertion is true would entail that p. But, the claim that every assertion is true doesn’t characterise our testimonial environment.)

KCS then attempt to defend factivity from the objection that it yields the counterintuitive result that a person and her BIV twin are not equally justified in believing that they have hands. The book criticised one standard infallibilist strategy for dealing with this data, namely to suggest that the BIV twin is merely excused for believing that she has hands and that we confuse excuse and justification. KCS defend this strategy claiming that the infallibilist is able to accommodate the intuitive data. They describe the dialectical situation as follows, “here is what we have so far: (1) the data to be explained – the positive intuition concerning the epistemic status of the BIV’s belief, and (2) the empirical data suggesting that we can’t discriminate between justification-triggered positive intuitions on the one hand and blamelessness-triggered positive intuitions on the other. If so, the data that needs to be accommodated… is that there is either justification or blamelessness present at BIV scenarios. New infallibilism does accommodate this data via the second leg of the disjunction”. KCS don’t offer any support for (2) and it just seems false. We routinely distinguish between morally justified and blameless action. Furthermore, we can distinguish epistemically justified and blameless belief, e.g. consider the case of a scientist who believes a claim which is supported by her evidence and a subject who believes a claim on the basis of a tempting but fallacious inference which she is unable to understand to be fallacious. So their description of the dialectic seems incorrect.

Having attempted to defend the key commitments of infallibilism, they raise some questions about the second half of the book in which I defend fallibilism against objections. One of those objections is that fallibilism violates closure for knowledge. In response, I argued that there is reason to suppose that closure fails quite independently of the dispute between fallibilism and infallibilism so that closure cannot be used to motivate infallibilism rather than fallibilism. The book focused on one way in which closure fails independently of that dispute, due to the phenomenon of defeat. In reply to such worries, KCS suggests that closure should be reformulated with a no-defeat condition. I agree that one could attempt to preserve closure in this way, but I suggest that it’s unmotivated to do so. The main motivation for closure is that we can clearly gain knowledge by valid inference from known premises. But, of course that we can often gain knowledge in this way is compatible with the failure of closure. In addition, there are other reasons to think that closure fails even on assumptions congenial to infallibilism. For instance, as Lasonen-Aarnio (2008) and Hawthorne and Lasonen-Aarnio (2009) argue, a safety condition on knowledge also leads to the failure of closure due to risk agglomeration even on a probability 1 view of knowledge.

Finally, KCS raise some worries about my discussion of the defeat solution to the dogmatism puzzle. Before addressing those worries, it’s worth noting that whether or not the defeat response to the dogmatism puzzle works is tangential to the main concerns of the book. The argument of the book does involve defending defeat. But I don’t do so by appealing to the defeat solution to the dogmatism puzzle.

The dogmatism puzzle arises from the fact that if a subject knows that p, then she also knows that any future evidence against p is misleading. But, then it’s puzzling why she isn’t simply entitled to disregard future evidence against p. One prominent response to this puzzle employs the notion of defeat: once one receives evidence against p, one’s prior knowledge that p is defeated so that one cannot employ that knowledge to dismiss the evidence against p. However, Lasonen-Aarnio points out that this leaves a residual worry in “no-defeat” cases in which one receives weak evidence that not-p which is not strong enough to defeat one’s knowledge that p. Intuitively, one should still reduce one’s credence but this cannot be explained by saying that one has lost one’s knowledge. A fallibilist may be tempted to say that, in such cases, once one receives evidence that not-p, this reduces the probability of p on one’s evidence so that one should reduce one’s credence but to a level compatible with knowing that p. But, Lasonen-Aarnio (2014) argued that that constituted a costly solution since the following kind of sentence doesn’t seem to be acceptable: “e is misleading evidence against p, but I ought to lower my credence in p as a result of having acquired e”.

In the book I offer several explanations of why such sentences may be intuitively unacceptable even if true. One factor that may be at work is that to say that something is misleading standardly conveys that one shouldn’t follow its lead. But, it’s a point of contention between defenders and opponents of the defeat solution whether having evidence that not-p when p, is misleading in the sense that one shouldn’t follow its lead. Defenders think that one should follow the lead of such evidence and no longer believe that p; opponents think one should retain the belief that p which still constitutes knowledge. Thus, I argued that in the context of this debate, use of the word “misleading” should be avoided in favour of the following more neutral description that one obtains evidence that not-p despite p being the case. If that’s right, then it’s not to the point to suggest, as KCS do, that “it is true in virtue of the meaning of “misleading” that if something is misleading, then one shouldn’t follow its lead”. I don’t dispute that there is a meaning of “misleading” in which that’s true. Rather, given that, I dispute whether it is legitimate to use “misleading” to describe the evidence in the dogmatism puzzle.

## Littlejohn

Littlejohn’s comments focus on my discussion of the knowledge view of justification, KVJ (a belief is justified if and only if it’s known). He sketches a number of positive arguments for KVJ, saying that he wishes I’d said more about why I think they fail. In addition, he offers an account of when a non-knowledge belief is excused which he hopes avoids the problems I raise for existing accounts. I consider these points in order.

One line of argument for KVJ which Littlejohn mentions combines the idea that knowledge is the norm for belief with the idea that justification is norm-conformity. Indeed, he expresses puzzlement how anybody could accept the knowledge norm of belief and yet not accept KVJ. I explained why I find this style of argument for KVJ unpersuasive in the book (93–94). There, I pointed out that even if an epistemologist accepts the controversial claim that knowledge is the norm of belief, she needn’t accept that the notion of justification in play in traditional epistemological debates is the notion of conformity to the norm of belief. Indeed, that doesn’t seem a charitable interpretation: many contemporary epistemologists accept a factive norm for belief (e.g. truth or knowledge) and at the same time have developed accounts of justification which allow that a false belief can be justified.

In reply, Littlejohn could stipulate that by “justification” he means conformity to the norm of belief but then a traditional epistemologist will think that he is simply changing the topic. Alternatively, he could suggest that epistemology doesn’t need the traditional notion of justification in addition to the notions of whether a belief conforms to the knowledge norm and whether it is excused. He suggests that if ethics can get by just with the notions of norm-conformity and excuse, perhaps epistemology can. If a defender of KVJ takes this second argumentative strategy, then she needs to show that there is no significant loss if epistemology jettisons the notion of justification. But, in the book, I argue that there would be a significant loss (94–95). The defender of KVJ has a hard time accommodating some of the important traditional roles of the notion of justification, especially its graded and propositional uses. Furthermore, without appeal to the notion of justification we seem to be left unable to make some of the important distinctions we want to make between non-knowledge beliefs. A traditional epistemologist can treat some of these beliefs as justified (e.g. belief in a false scientific hypothesis supported by the evidence) and some as merely excused (e.g. a subject who forms a belief via a tempting fallacy which she is in no position to understand to be fallacious). Notice that the scientist doesn’t need an excuse for her non-knowledge beliefs, and neither the scientist nor the fallacious thinker are exempt from the norms of belief. So, appeal to the knowledge norm, excuse and exemption doesn’t enable us to make all the distinctions we want as epistemologists.

A third potential style of argument for KVJ exploits alleged principles unifying the theoretical and practical. For instance, Littlejohn (2014) defends KVJ by appealing to the principle that the epistemic norms that govern practical and theoretical reasoning are unified in their demands (“Unity”). Combined with the further plausible idea that if one justifiably believes that p, then one is justified in believing the obvious consequences of p, this yields the claim that if you are justified in believing p, then you are justified in treating p as a reason for acting as if p. If one is already impressed with the idea that knowledge is the norm of practical reasoning, this might motivate identifying justification and knowledge. But, I have argued in detail against both Unity and the knowledge norm for practical reasoning (e.g. Brown 2008, 2012).

Let’s now turn to Littlejohn’s comments about excuse. Littlejohn questions why an infallibilist need offer an account of the conditions in which a non-knowledge belief is excused. Since the notion of an excusable norm violation is familiar from discussions in ethics, one might think that the infallibilist can simply exploit whatever is the correct account in ethics for her purposes. But, the infallibilist’s options are in fact much more constrained than this. For some popular claims in ethics about when one is excused for morally wrong action are unavailable to the defender of KVJ. For instance, some ethicists claim that justified false belief, or false belief that meets the norm of belief excuses moral wrongdoing. But the defender of KVJ denies that false beliefs can be justified or meet the norm of belief. Other ethicists claim that blameless false belief can excuse moral wrongdoing. But, this presupposes the notion of interest, what it is for a false belief to be blameless. In the book, I point out the unavailability of these options as well as criticise a range of “epistemic” accounts which attempt to provide a sufficient condition for excuse in terms of some epistemic condition (e.g. one isn’t in a position to know that one is violating the norm or has evidence supporting that one’s conforming to the norm). Thus, it’s no surprise that defenders of KVJ have offered dispositional and modal accounts of excuse of the kind I criticise in the book.

Littlejohn acknowledges the problems I raise for such dispositional accounts but attempts to overcome them in his expectation account. Dispositional accounts face two central problems. First, doing what most people disposed to conform to a norm would do isn’t sufficient for excuse. As parents frequently say, “That everybody else would do it doesn’t excuse your doing it!”. Second, I might do what most people disposed to conform to the norm would do in the circumstances, but do it for bad reasons (e.g. to hurt someone). Littlejohn attempts to get round both of these problems in his expectation account according to which an agent should be excused for wrongdoing if (1) she meets our expectations where “we meet expectations if our responses align with the responses of someone who is responsive, judicious, and handles risk well” in responding to right and wrong-making features and (2) “the reasoning behind [those responses] doesn’t show us to be unresponsive, less than judicious, or insensitive to risk”. Although this account is supposed to be an improvement on existing dispositional accounts, it still seems to appeal to a dispositional notion. In particular, given that excuse is needed only when a subject does wrong, in those circumstances she is not in fact responding appropriately to the right-and wrong-making features of the situation. So, condition (1) should be understood as saying that the agent’s response aligns with the response of someone who is disposed to be responsive, judicious, and risk-sensitive in responding to right- and wrong- making features. Thus by itself (1) faces the problems for dispositional accounts discussed above. Adding (2) doesn’t help. On similar grounds to (1), in (2) the notion of being responsive must be understood dispositionally. So, (2) says that the reasoning behind the agent’s response doesn’t show that the agent is not disposed to be responsive, judicious, and risk-sensitive in responding to right and wrong making features. But, compatibly with that, the agent might be blameworthy for wrongdoing. As Sher (2009) puts it, good people can do bad things, e.g. a kind person can intentionally make a hurtful remark on an occasion. Nor could one overcome this difficulty by strengthening the second condition to require that the agent doesn’t do wrong for the reasons which make it wrong. For, then, the condition wouldn’t be met by the BIV. She doesn’t, say, believe falsely that she has hands for the reason which makes that belief violate the knowledge norm (i.e. that it’s false). Thus, it seems to me that Littlejohn’s expectation account fails to provide a sufficient condition for excuse and that’s unsurprising in the light of Sher’s observation.

## Williamson

Williamson's comments raise some interesting issues about how we should do philosophy, as well as more specific points about some of my key objections to infallibilism. Before addressing the general methodological issues, let me reply to some of the more specific points he raises.

Let’s start by considering the problems I raise for the infallibilist’s commitment to SKSS (if one knows that p, then p is part of one’s evidence for p). It’s a problem for SKSS that when asked for one’s evidence for p, it’s infelicitous to cite p even if one knows that p. In his reply Williamson attempts to defend SKSS by appeal to the probability raising conception of evidential support. Like Greco he suggests that the infelicity data isn’t really a problem for infallibilism per se since the probability raising account of evidential support has the consequence that any proposition which is evidence is evidence for itself. And he suggests that to the extent that an alternative account of evidential support is offered which doesn’t have this consequence, the infallibilist could take advantage of it too. But, the latter suggestion ignores the broader dialectical situation. In particular, in the book I show how, on pain of accepting scepticism, infallibilism is committed to holding that if one knows that p, then p is part of one’s evidence for p. Infallibilism is committed to these claims since, without them, the evidence we have in support of a range of important knowledge claims doesn’t entail what’s known, including testimonial knowledge, knowledge by inference to the best explanation and knowledge by enumerative induction. By contrast, since the fallibilist denies that knowledge requires entailing evidence, she needn’t make these commitments. Thus, infallibilism cannot be satisfactorily defended by appeal to a conception of evidential support which doesn’t support SKSS.

So, let’s turn to the suggestion that one can support SKSS by appeal to the probability raising conception of evidential support. The probability raising conception is controversial. Furthermore, just appealing to that conception doesn’t offer any explanation of the infelicity of citing a proposition as evidence for itself. So, an infallibilist who endorses the probably raising conception also needs to explain the infelicity of citing a known proposition as evidence for itself. On the face of it, there are three main potential explanations: pragmatic explanation, an error theoretic explanation, or appeal to contextualism. In the book and elsewhere (e.g. 2015) I argue against Williamson’s (2000) pragmatic explanation as well as error theoretic and contextualist explanations.

In principle, a different kind of reply might seem available, the kind of move standardly used to defend the formal logical definition of “validity” in the face of worries that it has the result that certain arguments get counted as valid even if they would not ordinarily be judged so (e.g. the argument: p therefore p). The logician may simply be interested in a technical sense of “valid” that is not coextensive with its ordinary use. It’s no objection to this technical use that it doesn’t accord with the way we ordinarily speak. Williamson suggests in his reply that a similar “ambiguity” response may be used to defend the probability raising conception of evidential support. While he doesn’t commit to this option, he says “we do not need to decide between the ambiguity and error approaches here, since they serve present purposes equally well”.

I agree that it’s open to a formal epistemologist to say that she is interested in notions of “evidence” and “evidential support” which are technically defined and not coextensive with the ordinary senses of these expressions. And I have no complaint against this as such. But, to the extent that this is the way the probability raising conception of evidential support is defended, it’s not clear how it connects with the epistemological controversies concerning infallibilism to which the book is a contribution. As the book explains, the choice between fallibilism and infallibilism lies at the heart of important debates concerning the nature and extent of knowledge and evidence, the connection between knowledge, assertion and practical reasoning, and our ability to extend our knowledge through argument. Furthermore, many of the key arguments draw on our ordinary ways of thinking and talking about knowledge and evidence. In particular, infallibilists have challenged fallibilism by appeal to the infelicity of concessive knowledge attributions, the infelicity of claiming to know lottery propositions, as well as the ordinary ways in which we criticise and defend assertions and practical reasoning. To the extent that the infallibilist says that she is interested in technical notions of evidence, knowledge, and evidential support, it’s not clear to what extent she is engaging with much of the contemporary philosophical literature around infallibilism. Furthermore, it’s not clear to what extent she is entitled to appeal to these ordinary ways of speaking and talking in defending her own position.

Now let’s turn to the broad methodological points which Williamson raises. Williamson endorses an abductive methodology according to which we should compare fully fleshed out rival theories with respect to their abductive virtues: fit with evidence, simplicity, elegance, informativeness and explanatory power. He worries that, by contrast, my book instead involves comparing “one worked-out theory, infallibilism… with a much sketchier version of fallibilism”. But, he suggests that this makes my life too easy by appeal to the Kuhnian point that any scientific paradigm at any time has unresolved anomalies. He suggests that it’s illegitimate to press these worries before one has an alternative fully-fleshed out rival theory. I take it that, in a variety of ways, the book does serve to flesh out fallibilism, even though I fully accept that further work needs to be done. But even setting aside the issue of whether the fallibilism I defend is fully-fleshed out, it seems to me, contra Williamson, that one can usefully make progress by pointing out defects in an existing theory, even before having a fully-fleshed out rival theory. Even if one doesn’t have a fully-fleshed out rival theory to hand, that doesn’t make problems for an existing theory any less problematic. Pointing out these defects is often a useful way of either refining and developing the existing theory or developing a rival theory. If evaluation of one theory always needed to wait until we had a fully fleshed-out rival theory, this would be a hindrance rather than a help to investigation, whether philosophical or scientific.
